# Dr. Frank Gamon

**Published:** 1917-04

**Authors:** 


**Dr. Frank Gamon,** Medico-Chirurgical of Philadelphia 1903, a practitioner of Erie, Pa., died at the Hamot Hospital, Dec. 23, 1916, aged 36.

